# Long non-coding ROR promotes the progression of papillary thyroid carcinoma through regulation of the TESC/ALDH1A1/TUBB3/PTEN axis

**DOI:** 10.1038/s41419-021-04210-9

**Published:** 2022-02-16

**Authors:** Yuxia Fan, Xin Fan, Hao Yan, Zheng Liu, Xiaoming Wang, Qingling Yuan, Jie Xie, Xiubo Lu, Yang Yang

**Affiliations:** 1 Department of Thyroid Surgery, The First Affiliated Hospital of Zhengzhou University, Zhengzhou, 450000 P.R. China; 2 Department of Urology Surgery, The First Affiliated Hospital of Zhengzhou University, Zhengzhou, 450000 P.R. China; 3 Department of Thoracic Surgery, The First Affiliated Hospital of Zhengzhou University, Zhengzhou, 450000 P.R. China

**Keywords:** Gynaecological cancer, Cell biology

## Abstract

Papillary thyroidal carcinoma (PTC) is a common endocrine cancer that plagues people across the world. The potential roles of long non-coding RNAs (lncRNAs) in PTC have gained increasing attention. In this study, we aimed to explore whether lncRNA ROR affects the progression of PTC, with the involvement of tescalcin (TESC)/aldehyde dehydrogenase isoform 1A1 (ALDH1A1)/βIII-tubulin (TUBB3)/tensin homolog (PTEN) axis. PTC tumor and adjacent tissues were obtained, followed by measurement of lncRNA ROR and TESC, ALDH1A1, and TUBB3 expression. Interactions among lncRNA ROR, TESC, ALDH1A1, TUBB3, and PTEN were evaluated by ChIP assay, RT-qPCR, or western blot analysis. After ectopic expression and depletion experiments in PTC cells, MTT and colony formation assay, Transwell assay, and flow cytometry were performed to detect cell viability and colony formation, cell migration and invasion, and apoptosis, respectively. In addition, xenograft in nude mice was performed to test the effects of lncRNA ROR and PTEN on tumor growth in PTC in vivo. LncRNA ROR, TESC, ALDH1A1, and TUBB3 were highly expressed in PTC tissues and cells. Overexpression of lncRNA ROR activated TESC by inhibiting the G9a recruitment on the promoter of TESC and histone H3-lysine 9me methylation. Moreover, TESC upregulated ALDH1A1 expression to increase TUBB3 expression, which then reduced PTEN expression. Overexpression of lncRNA ROR, TESC, ALDH1A1 or TUBB3 and silencing of PTEN promoted PTC cell viability, colony formation, migration, and invasion while suppressing apoptosis. Moreover, overexpression of lncRNA ROR increased tumor growth by inhibiting PTEN in vivo. Taken together, the current study demonstrated that lncRNA ROR mediated TESC/ALDH1A1/TUBB3/PTEN axis, thereby facilitating the development of PTC.

## Introduction

Papillary thyroidal carcinoma (PTC) is the major type of thyroid cancer that is prevalent across the world [1]. The incidence of PTC has continued to increase in the past few decades [2]. Histopathologically, PTC is normally well-differentiated and displays multiple histological variants [3]. It is usually multifocal, and characterized by intraglandular metastasis from a single primary tumor or co-existence of diverse primary tumors [4]. The main risk factors of PTC comprise ionizing radiation and family history [5]. The first-line treatment option for patients with thyroid cancer is considered to be surgery if eligible, while patients with PTC resected are vulnerable to a recurrence in the neck [6]. Notably, long non-coding RNAs (lncRNAs) have been reported to show aberrant expression in PTC [7], which renders increasing attention to the roles of lncRNAs in PTC.

LncRNAs, a subclass of non-coding RNAs that contain more than 200 nucleotides, have been found as key regulators in cancer development and progression [8]. LncRNA regulator of reprogramming (ROR), a mediator of reprogramming, is regarded as an oncogene that participates in cancer development [9]. Interestingly, a previous study has reported the crucial regulatory roles of lncRNA ROR in the progression of PTC [10]. Moreover, existing literature has shown that lncRNA ROR facilitates tumor progression by upregulating tescalcin (TESC) expression [11]. TESC is a 24-kDa EF-hand Ca^2+^-binding protein capable of regulating differentiation as well as cell growth [12]. TESC has been suggested as a potential irradiation-induced biomarker for PTC [13]. Furthermore, TESC has been reported to promote lung cancer stemness by upregulating the expression of aldehyde dehydrogenase isoform 1A1 (ALDH1A1) [14], a gene that is considered a predictor of poor prognosis for patients with PTC [15]. It is also worthy to note that ALDH1A1 contributes to bladder cancer progression by increasing the expression of βIII-tubulin (TUBB3) [16]. TUBB3 has been previously reported to facilitate tumorigenesis of non-small cell lung cancer through the PTEN/AKT axis [17]. Interestingly, loss of PTEN is frequently identified in follicular variant of PTC [18]. Therefore, considering the aforementioned reports, we hypothesized that lncRNA ROR is likely to affect the development of PTC through regulation of the TESC/ALDH1A1/TUBB3/PTEN axis. By testing this hypothesis in this study, we hope to identify a novel target for PTC treatment.

## Materials and methods

### Ethics statement

The current study was approved by the Ethics Committee of The First Affiliated Hospital of Zhengzhou University and conducted in strict accordance with the Declaration of Helsinki. All participants signed informed consent documentation before sample collection. Animal experiments were approved by the Animal Ethics Committee of The First Affiliated Hospital of Zhengzhou University and performed strictly following the Guide for the Care and Use of Laboratory Animals published by the US National Institutes of Health.

### Bioinformatics prediction

The downstream gene of lncRNA ROR was identified through existing literature. Differential expression analysis was conducted on thyroid cancer datasets GSE3467, GSE33630, and GSE97001 in the GEO database (https://www.ncbi.nlm.nih.gov/gds) using R language “limma” package (http://www.bioconductor.org/packages/release/bioc/html/limma.html), with |logFoldChange| > 1.3 and *p* < 0.01 as the thresholds. There are 18 samples in total in the GSE3467 dataset, including 9 normal samples and 9 thyroid cancer samples; 105 samples in the GSE33630 dataset, including 45 normal samples and 60 thyroid cancer samples; 8 samples in the GSE97001 dataset, including 4 normal samples and 4 thyroid cancer samples. Gene co-expression analysis was carried out through the MEM (https://bit.cs.ut.ee/mem/index.cgi) database, and GeneMANIA (http://genemania.org) was applied to construct PPI network of genes, among which those with higher core degree were selected for research. Through the online analysis tool UALCAN (http://ualcan.path.uab.edu/index.html), the data of TCGA database (https://portal.gdc.cancer.gov/) were analyzed and the expression trend of the relevant genes was obtained. Finally, PanCancer module of Starbase (starbase.sysu.edu.cn) was used to perform the correlation analysis of gene expression.

### Study subjects

A total of 85 tumor samples from patients with PTC who underwent radical resection at The First Affiliated Hospital of Zhengzhou University between March 2019 and December 2019, as well as adjacent tissues were selected for this study. None of these patients received chemotherapy or radiotherapy prior to this study. Patients with other tumors or cancer history were excluded from this study. The clinical information of patients is shown in Table S1. The total sample was dissected immediately after sample collection and placed on ice. The sample was frozen rapidly with liquid nitrogen and stored at −80 °C for subsequent use.

### Immunohistochemistry

Specimens were taken out after fixed with 10% formaldehyde, paraffin-embedded, and prepared into 4-μm serial sections. Tissue sections were incubated in 3% H_2_O_2_ (Sigma-Aldrich Chemical Company, St Louis, MO, USA) at 37 °C for 30 min, rinsed with phosphate buffer saline (PBS), and then boiled at 95 °C for 20 min in 0.01 M citric acid buffer. After cooling down to room temperature, the sections were sealed with normal goat serum working fluid at 37 °C for 10 min. Primary rabbit anti-rat antibodies, including TESC (ab103695, 1:100), ALDH1A1 (ab227948, 1:100), and TUBB3 (ab18207, 1:100), were added to the sections for 2-h incubation at 37 °C. Following a PBS rinse, HRP-labeled secondary goat anti-rabbit IgG working solution (ab6721, 1:1000) was co-incubated with the sections in a wet box at 37 °C for 30 min. Both the primary and secondary antibodies were provided by Abcam Inc. (Cambridge, UK). Hematoxylin (Shanghai Fu Sheng Industrial Co., Ltd, Shanghai, China) was applied to the sections at room temperature. After the excessive staining solution was rinsed off under running water, the sections were fixed with 10% glycerol/PBS and observed under a microscope. The results were separately scored by two experienced pathologists using the double-blind method, and analyzed using ImageJ software.

### Cell treatment

Human normal thyroid cell line Nthy-ori 3-1 (Shanghai EK-Bioscience, CC-Y1708, http://www.elisakits.cn/Index/product/ccid/147.html) and PTC cell lines TPC-1 (EK-Bioscience, CC-Y1522) were cultured in RPMI 1640 medium (12633012, GIBCO) containing 10% fetal bovine serum (FBS) and 1% P/S. PTC cell lines BCPAP (EK-Bioscience, CC-Y1064) and IHH4 (Shanghai YaJi Biological, YS1398C) were cultured in Dulbecco’s modified Eagle’s medium (DMEM) (11965092, GIBCO) containing 10% fetal bovine serum (FBS) and 1% P/S.

Lentiviral vectors LV5-GFP (used for gene overexpressing; Addgene, Cambridge, MA, USA, #25999) and pSIH1-H1-copGFP (used for gene silencing, BD Biosciences, Franklin Lakes, NJ, USA, LV601B-1) were used to generate viral particles for transducing PTC cells. The cells received no treatment; or transduced with LV5-oe-NC, LV5-oe-lncRNA ROR, LV5-oe-TESC, LV5-oe-ALDH1A1, LV5-oe-TUBB3, pSIH1-H1-sh-NC, pSIH1-H1-sh-ROR-1, pSIH1-H1-sh-ROR-2, pSIH1-H1-sh-TESC-1, pSIH1-H1-sh-TESC-2, pSIH1-H1-sh-ALDH1A1-1, pSIH1-H1-sh-ALDH1A1-2, pSIH1-H1-sh-TUBB3-1, or pSIH1-H1-sh-TUBB3-2; or co-transduced with LV5-oe-lncRNA ROR and pSIH1-H1-sh-TESC-1, LV5-oe-lncRNA ROR and pSIH1-H1-sh-ALDH1A1-1, LV5-oe-ALDH1A1 and pSIH1-H1-sh-TUBB3-1, LV5-oe-TUBB3 and LV5-oe-PTEN, or LV5-oe-lncRNA ROR and LV5-oe-PTEN. The lentivirus was packaged with 293 T cells, which were cultured in Roswell Park Memorial Institute-1640 (RPMI-1640) complete medium containing 10% FBS and passaged every other day. The virus (1 × 10^8^ TU/mL) was added to the PTC cell lines for transduction and subsequent treatment.

### RT-qPCR

Total RNA was extracted using TRIzol reagent (15596026, Invitrogen, Carlsbad, CA, USA). A RT kit (RR047A, Takara, Otsu, Shiga, Japan) was applied to reversely transcribe the RNA into complementary DNA (cDNA). Samples were loaded using a SYBR Premix EX Taq kit (RR420A, Takara) and then subjected to qPCR on a real-time PCR machine (ABI7500, ABI Company, Oyster Bay, NY). The primers of lncRNA ROR, TESC, ALDH1A1, TUBB3, and PTEN were synthesized by Shanghai Sangon Biotechnology Co. Ltd, (Shanghai, China) (Table S2). Glyceraldehyde-3-phosphate dehydrogenase (GAPDH) and U6 were used as the internal reference. Fold changes in gene expression were calculated using relative quantification (2^−^^ΔΔCt^ method).

### Western blot analysis

High-efficiency radio-immunoprecipitation assay lysis buffer (R0010, Solarbio, Beijing, China) was used to extract total protein from tissues or cells, in strict accordance with the instructions. After lysis for 15 min at 4 °C, centrifugation was conducted at 15,000 r/min for 15 min, and the supernatant was extracted. Next, the protein concentration of each sample was determined using a bicinchoninic acid kit (20201ES76, Yeasen Company, Shanghai, China). After protein isolation by polyacrylamide gel electrophoresis, the protein was transferred to a polyvinylidene fluoride membrane by wet transfer method, and sealed with 5% bovine serum albumin for 1 h at room temperature. Following three Tris-buffered saline Tween-20 (TBST) washes, 5 min each, diluted primary rabbit anti-human antibodies to TESC (ab103695, 1:500), ALDH1A1 (ab227948, 1:1000), TUBB3 (ab18207, 1:500), and PTEN (ab31392, 1:1000), which were all purchased from Abcam Inc. (Cambridge, UK), were added to the membrane, followed by overnight incubation at 4 °C. Following an additional three TBST rinses at 5 min each, horseradish peroxidase (HRP)-labeled goat anti-rabbit to immunoglobulin G (IgG) diluent (ab205718, 1:10,000, Abcam) was added to the membrane for 1-h incubation at room temperature. After that, the membrane was developed using VILBER FUSION FX5 (Vilber Lourmat, Marne La Valle´e, France). With the use of the ImageJ 1.48u software (National Institutes of Health, Bethesda, Maryland, USA), the gray ratio of each protein to the internal reference GAPDH was used for protein quantitative analysis.

### Colony formation assay

The experiment was prepared when cells reached 70% confluence and the culture medium was renewed the night before the experiment. Two parts of 1 g agarose gel of low melting point was put into fresh-prepared MiliQ water to make 10% and 2% mixtures, respectively. Next, the mixtures were sterilized under high pressure and kept at 40 °C, avoiding cooling and solidifying. A small amount of RPMI-1640 medium containing 10% FBS was preheated to 37 °C. Next, 10% gel solution of low melting point maintained at 40 °C was mixed with the preheated RPMI-1640 medium at a ratio of 10:1. A total of 5 mL of the mixture was put into a sterile culture dish (6 cm in diameter), cooled down, solidified, and reserved in a 37 °C incubator with 5% CO_2_. Cells in culture were prepared into single cells with 0.25% trypsin and centrifuged at 200 × *g* and 20 °C for 15 min, with the supernatant subsequently gently poured out. Cells were triturated and suspended with a small amount of 10% FBS plus RPMI-1640, counted, and diluted to 500 cells/mL. Next, 2% gel solution of low melting point maintained at 40 °C was sufficiently mixed with the cell suspension at a ratio of 10:1, and then 2 mL of the mixture was placed onto the bottom of the prepared agarose. After the added agarose was coagulated, it was placed into a 37 °C incubator with 5% CO_2_. Subsequently, 2% gel solution of low melting point maintained at 40 °C was sufficiently mixed together with 10% FBS plus RPMI-1640 at a ratio of 10:1, and then 1 mL of the mixture was placed onto the bottom of the prepared agarose. After the added agarose was coagulated, 5 mL of 10% FBS plus RPMI-1640 was added to the agarose, the mixture of which was cultured in a 37 °C incubator with 5% CO_2_ for 7 days. The transformed TPC-1 cells were divided into three parts. The colony formation rate was observed, and counted as follows: colony formation rate = the number of colonies/the number of inoculated cells.

### MTT assay

The culture medium containing 10% FBS was used to prepare a single-cell suspension, which was inoculated into a 96-well plate at a density of 1000–10,000 cells per well, with a volume of 200 μL. Cells were routinely cultured for 3–5 days, after which 20 μL MTT solution (prepared with 5 mg/mL PBS) was added to each well for further incubation. After 4 h, the incubation was terminated, the supernatant was removed carefully, and centrifugation was performed for suspended cells before removal of the supernatant inside the well. Next, 150 μL dimethyl sulfoxide was added to each well, followed by shaking for 10 min in order to fully dissolve the crystals. The optical density of each well was measured at 490 nm wavelength using enzyme-linked immunosorbent assay, and the results were recorded. The growth curve was drawn with time as *X*-axis and optical density as *Y*-axis.

### Chromatin immunoprecipitation

PTC cells were fixed with formaldehyde for 10 min to induce DNA-protein crosslinking. An ultrasound crusher (UP-250, Scientz, Ningbo, China) was used to disrupt the cells into fragments for a total of 15 cycles, 10 s each at intervals of 10 s. The supernatant was collected through centrifugation at 4 °C and 12,000 r.p.m. for 10 min and placed into two separate tubes. NC rabbit antibodies to IgG (ab109489, 1:300), G9a (1:100, ab40542), and H3K9 methylation (#4473, 1:100, Cell Signaling Technologies, Beverly, MA, USA) were, respectively, added to the tubes and incubated overnight at 4 °C to for full binding purpose. These antibodies were purchased from Abcam. DNA–protein complexes were precipitated using Protein Agarose/Sepharose, and the supernatant was discarded after centrifugation at 12,000 × *g* for 5 min. The non-specific complexes were washed, followed by de-crosslinking overnight at 65 °C. The DNA fragments were then purified via phenol/chloroform extraction the next day. The conditions of PCR (3 μL in liquid wax) contained 2 μL chromatin immunoprecipitation (ChIP) (or Input) DNA, 0.5 mM appropriate primer pairs, 50 μM deoxynucleotide triphosphate, and 0.2 U Klen-Taq I (Ab Peptides, St Louis, MO, USA). Standard PCR was performed using the ABI Prism 7500 Sequence Detection System (USA) and Power SYBR®Green PCR Master Mix (Applied Biosystems, Carlsbad, CA, USA). The primers are depicted in Table S3.

### Transwell migration and Matrigel invasion assays

Cell migration and invasion in vitro were detected using Transwell chambers (well diameter of 8 µm; Corning, Corning, NY, USA) in a 24-well plate. Before the exploration, 600 µL DMEM containing 20% FBS was added in plycarbonate film Transwell chambers with Matrigel or Transwell chambers without Matrigel for 1-h equilibrium at 37 °C. PTC cells after transduction for 48 h were re-suspended in DMEM containing 10% FBS. The 100 µL cell suspension (1 × 10^9^ cells/L) was added into the apical chambers for 24-h culture at 37 °C with 5% CO_2_. Transwell chambers were then taken out and cells on the inner side of microwell membranes were wiped out using cotton swab. After a PBS wash, cells were fixed with 4% methanol, stained with 0.1% crystal violet, observed, and photographed under an inverted microscope. Five fields were selected on a random basis.

### Flow cytometry

Cells were washed twice with PBS, centrifuged, and dispersed into single-cell suspension (5 × 10^5^ cells/mL) using binding buffer. Then, 5 µL Annexin V was added at room temperature under conditions void of light, followed by addition of 5 µL 7-amino-actinomycin D. After culture for 30 min, cell apoptosis was detected by a flow cytometer (BD Company, San Jose, CA, USA).

### Xenograft in nude mice

A total of 48 BALB/c nude mice (aged 5–7 weeks and weighing 17–22 g; purchased from Beijing Vital River Laboratory Animal Technology Co., Ltd, Beijing, China, 401) were selected. Animal experiments were conducted in Institute of Medicine, University of Zhengzhou, Henan Province (Zhengzhou, Henan, China). The mice were housed under the specific-pathogen-free environment. They received adaptive feeding for 7 days under the comfortable temperature environment, with sterile food and drinking water under an alternative 12-h day/night cycle. The above nude mice were engrafted with different cells: non-transduced cells (wild-type [WT] mice), or cells transduced with lentivirus carrying oe-NC, oe-lncRNA ROR, or oe-lncRNA ROR + oe-PTEN.

The cells growing in the logarithmic phase after transduction were prepared into 5 × 10^7^ cells/mL cell suspension, and 0.2 mL suspension was subcutaneously injected into the left axillary region of the BALB/c nude mice with a 1 mL syringe. After inoculation, all nude mice were kept in a laminar flow hood in a specific-pathogen-free animal house. The tumor growth was observed every 5 days, and the data were recorded for 30 days. The long diameter and short diameter of the tumors were recorded with a ruler. The volume of tumors was calculated according to the formula: volume = long diameter × short diameter^2^/2. Meanwhile, the weight of tumors was weighed using a balance.

### Statistical analysis

Data analysis was performed using SPSS 21.0 (IBM Corp., Armonk, NY, USA). Measurement data were presented as mean ± standard deviation. Unpaired *t*-test was utilized to compare the unpaired data between two groups conforming to normal distribution and homogeneity of variance. Comparisons among multiple groups were analyzed using one-way analysis of variance (ANOVA), with Tukey’s test performed for post hoc test. Comparisons among multiple groups at different time points were analyzed by repeated measures ANOVA, followed by Bonferroni post hoc test. The survival rates of the patients were calculated by the Kaplan–Meier method, and the log-rank test was used for univariate survival analysis. A value of *p* < 0.05 indicated a statistically significant difference.

## Results

### LncRNA ROR was highly expressed in PTC tissues and cells while its silencing suppressed PTC cell proliferation, migration, and invasion while promoting cell apoptosis

**Fig. 1:**
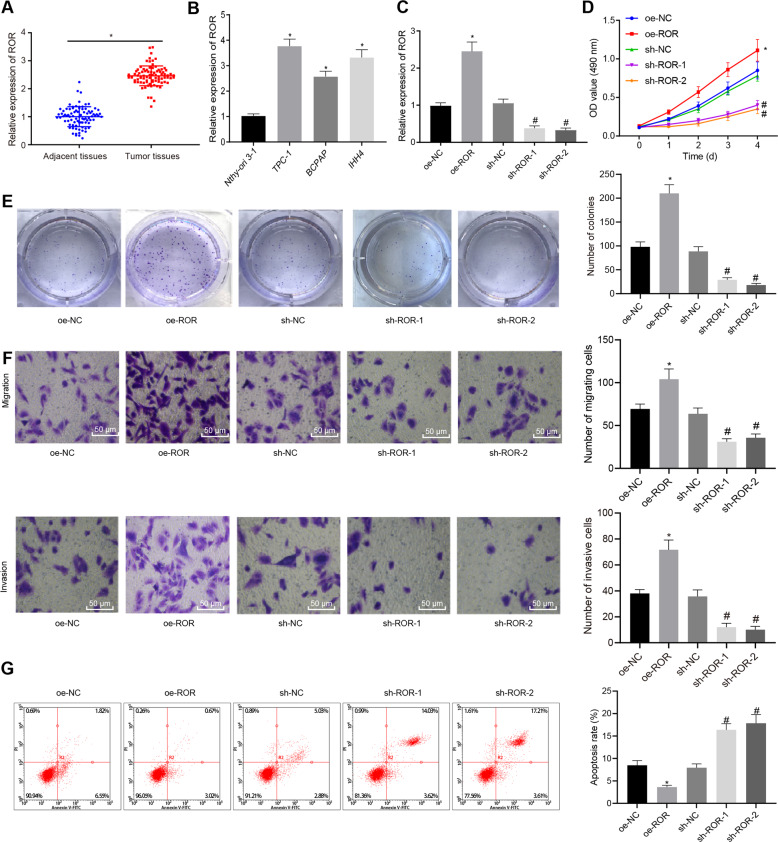
LncRNA ROR is upregulated in PTC tissues and cells and promotes features associated to malignancy in TPC-1 cells. **A** LncRNA ROR expression in PTC tissue and adjacent tissue samples of patients with PTC as detected by RT-qPCR, normalized to GAPDH (*n* = 85). *p < 0.05 vs. adjacent tissues. **B** LncRNA ROR expression in PTC cell lines TPC-1, BCPAP, IHH4, as well as normal thyroid Nthy-ori 3-1 cell line as detected by RT-qPCR, normalized to GAPDH. *p < 0.05 vs. Nthy-ori 3-1. **C** LncRNA ROR overexpression and silencing efficiency in TPC-1 cells verified by RT-qPCR. **D** TPC-1 cell viability after alteration of lncRNA ROR as detected by MTT. **E** Size and number of colonies in TPC-1 cells after alteration of lncRNA ROR, as detected by colony formation assay. **F** TPC-1 cell migration and invasion after alteration of lncRNA ROR as detected by Transwell assay (×200). **G** TPC-1 cell apoptosis after alteration of lncRNA ROR as detected by flow cytometry. *p < 0.05 vs. Nthy-ori 3-1 cells or oe-NC, ^#^*p* < 0.05 vs. sh-NC. Cell experiments were repeated three times independently.

LncRNA ROR has been found to regulate pancreatic cancer, esophageal squamous cell carcinoma, and other cancers, and it is highly expressed in PTC [10, 19, 20]. Here we collected 85 cases of PTC and adjacent tissues from patients with PTC. Through reverse transcription and quantitative polymerase chain reaction (RT-qPCR), lncRNA ROR was found to be highly expressed in PTC tissues (Fig. 1A). Then, we analyzed lncRNA ROR expression using RT-qPCR in several PTC cell lines including TPC-1, BCPAP, IHH4, as well as normal thyroid Nthy-ori 3-1 cell line (Fig. 1B). The results revealed that lncRNA ROR expression remarkably increased in TPC-1, BCPAP, and IHH4 cell lines versus that in Nthy-ori 3-1 cell line.

To detect the effect of lncRNA ROR on PTC, lncRNA ROR was overexpressed or silenced in TPC-1 and BCPAP cell lines through lentivirus-mediated gene manipulation. The overexpression and silencing efficiency of lncRNA ROR in TPC-1 and BCPAP cells was verified by RT-qPCR (Fig. 1C and Fig. S1A). The PTC cell proliferation was analyzed by 3-(4,5-dimethylthiazol-2-yl)-2,5-diphenyltetrazolium bromide (MTT) and cell colony formation assay (Fig. 1D, E and Fig. S1B, C), cell migration and invasion by Transwell assay (Fig. 1F and Fig. S1D), and cell apoptosis by flow cytometry (Fig. 1G and Fig. S1E). The cell viability, size and number of colonies, migration, and invasion increased significantly in response to lentivirus-mediated overexpression of lncRNA ROR, but decreased by short hairpin RNA (sh)-lncRNA ROR-1 or sh-lncRNA ROR-2. The cell apoptosis turned out to change in an opposite manner. The above experiments fully demonstrated that lncRNA ROR promoted features associated to malignancy in PTC cells.

### LncRNA ROR upregulated TESC expression by inhibiting G9a recruitment on the TESC promoter and H3K9me methylation

**Fig. 2:**
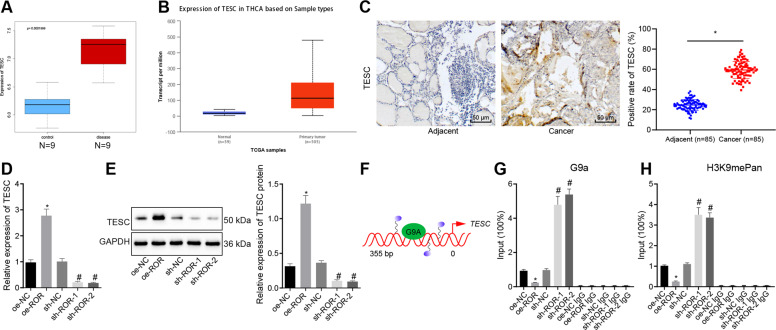
LncRNA ROR increases TESC expression by repressing the recruitment of G9a on the TESC promoter and H3K9me methylation in TPC-1 cells. **A** The TESC expression box line diagram drawn based on the GSE3467 dataset. The blue box on the left represents the expression of normal samples (*n* = 9), and the red box on the right represents the expression of tumor samples (*n* = 9). **B** The TESC expression box line diagram drawn based on data in the TCGA database following UALCAN analysis. The blue box on the left represents the expression of normal samples, and the red box on the right represents the expression of tumor samples. **C** TESC expression in PTC tissue and adjacent tissue samples of patients with PTC by immunohistochemistry (×400; *n* = 85). *p < 0.05 vs. adjacent tissues. **D** TESC expression after alteration of lncRNA ROR in TPC-1 cells as determined by RT-qPCR, normalized to GAPDH. **E** TESC protein expression after alteration of lncRNA ROR in TPC-1 cells as determined by western blot analysis, normalized to GAPDH. **F** Schematic diagram of the binding of G9a to the TESC promoter. **G** G9a recruitment on the promoter of TESC after alteration of lncRNA ROR in TPC-1 cells, as measured by ChIP-PCR. **H** H3K9me methylation of the TESC promoter after alteration of lncRNA ROR in TPC-1 cells, as detected by ChIP-PCR. In panels **D**, **H**, *p < 0.05 vs. oe-NC, ^#^*p* < 0.05 vs. sh-NC. Cell experiments were repeated three times independently.

The box line diagram drawn, based on TESC expression data from GE3467 dataset in the Gene Expression Omnibus (GEO) database, showed that TESC was highly expressed in thyroid cancer samples (Fig. 2A), and similar results were seen in the thyroid cancer expression datasets from The Cancer Genome Atlas (TCGA) using the UALCAN database (http://ualcan.path.uab.edu/index.html) (Fig. 2B). To investigate the relationship of lncRNA ROR and TESC in PTC, we first analyzed the expression of TESC in PTC tissue samples of patients with PTC and adjacent tissues by immunohistochemistry. Compared to adjacent tissues, TESC expression in PTC tissues was significantly increased (Fig. 2C). Next, TESC expression in TPC-1 and BCPAP cells was determined by RT-qPCR and western blot analysis after overexpression or silencing of lncRNA ROR (Fig. 2D, E and Fig. S2A, B). The results showed that TESC expression was markedly upregulated by overexpression (oe)-lncRNA ROR, but downregulated by sh-lncRNA ROR-1 or sh-lncRNA ROR-2.

Existing literature has revealed that lncRNA ROR occupies and activates the TESC promoter by repelling histone methyltransferase G9a and promoting the release of histone H3K9 methylation to promote cancer [11]. Therefore, we speculated that lncRNA ROR may also regulate the expression of TESC by affecting the binding of G9a protein to the TESC promoter in PTC cells. For verification, ChIP-PCR analysis was first used to detect the binding of G9a on the TESC promoter in TPC-1 and BCPAP cells. The results revealed that G9a recruitment on the TESC promoter was significantly decreased upon overexpression of lncRNA ROR. On the contrary, G9a recruitment on the TESC promoter was significantly increased when cells treated with sh-lncRNA ROR-1 or sh-lncRNA ROR-2 was used (Fig. 2F, G and Fig. S2C). This result indicated that overexpression of lncRNA ROR may inhibit the interaction between G9a protein and the TESC promoter, while knockdown of lncRNA ROR promoted the interaction between G9a protein and the TESC promoter. Subsequently, we used ChIP-PCR to detect H3K9me methylation status of the TESC promoter in TPC-1 and BCPAP cells, with the results showing that the methylation level of H3K9me had a notable decline upon overexpression of lncRNA ROR but a notable elevation was noted upon silencing of lncRNA ROR-1 (Fig. 2H and Fig. S2D). Collectively, lncRNA ROR may promote TESC expression by inhibiting the recruitment of G9a on the TESC promoter and the methylation of H3K9me.

### LncRNA ROR promoted PTC cell proliferation, migration, and invasion while inhibiting cell apoptosis by upregulating ALDH1A1 through TESC

**Fig. 3:**
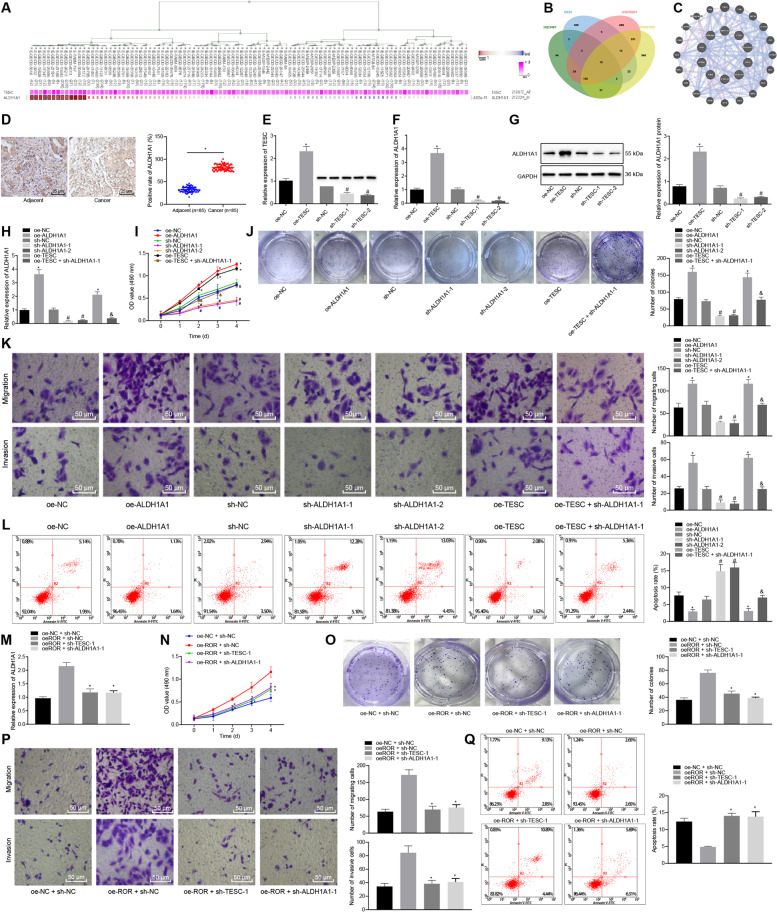
LncRNA ROR upregulates ALDH1A1/TESC to promote TPC-1 cell proliferation, migration, and invasion while inhibiting cell apoptosis. **A** The co-expression relationship between TESC and ALDH1A1 from MEM analysis. ALDH1A1 has multiple probes, i.e., multiple co-expression relationships are shown in the figure. **B** Venn diagram of differentially expressed genes in thyroid cancer samples from the GSE3467, GSE33630, and GSE97001 datasets and co-expression genes predicted using MEM. **C** PPI network diagram of 10 intersection genes and their related genes constructed by GeneMANIA. The larger the circle for the gene is, the higher the core degree is. **D** ALDH1A1 expression in PTC tissue and adjacent tissue samples of patients with PTC as detected by immunohistochemistry (×400, *n* = 85). *p < 0.05 vs. adjacent tissues. **E** TESC expression after alteration of TESC in TPC-1 cells as determined by RT-qPCR, normalized to GAPDH. *p < 0.05 vs. oe-NC. ^#^*p* < 0.05 vs. sh-NC. **F** ALDH1A expression after alteration of TESC in TPC-1 cells as determined by RT-qPCR, normalized to GAPDH. *p < 0.05 vs. oe-NC. ^#^*p* < 0.05 vs. sh-NC. **G** ALDH1A protein expression after alteration of TESC in TPC-1 cells as determined by western blot analysis, normalized to GAPDH. *p < 0.05 vs. oe-NC. ^#^*p* < 0.05 vs. sh-NC. **H** ALDH1A1 ex*p*ression after ALDH1A1 alteration and TESC overexpression in TPC-1 cells as determined by RT-qPCR. **I** TPC-1 cell viability after ALDH1A1 alteration and TESC overexpression, as detected by MTT. **J** Colony formation of TPC-1 cells after ALDH1A1 alteration and TESC overexpression, as detected by colony formation assay. **K** TPC-1 cell migration and invasion after ALDH1A1 alteration and TESC overexpression as detected by Transwell assay (×200). **L** TPC-1 cell apoptosis after ALDH1A1 alteration and TESC overexpression as detected by flow cytometry. In panel **H**–**L**, *p < 0.05 vs. oe-NC, ^#^*p* < 0.05 vs. sh-NC, ^&^*p* < 0.05 vs. oe-TESC. **M** The expression of ALDH1A1 in TPC-1 cells transduced with oe-lncRNA ROR + sh-TESC-1 or sh-ALDH1A1-1, as determined by RT-qPCR, normalized to GAPDH. **N** TPC-1 cell viability after transduction with oe-lncRNA ROR + sh-TESC-1 or sh-ALDH1A1-1, as detected by MTT. **O** Colony formation of TPC-1 cells after transduction with oe-lncRNA ROR + sh-TESC-1 or sh-ALDH1A1-1, as detected by colony formation assay. **P** TPC-1 cell migration and invasion after transduction with oe-lncRNA ROR + sh-TESC-1 or sh-ALDH1A1-1 as detected by Transwell assay (×200). **Q** TPC-1 cell apoptosis after transduction with oe-lncRNA ROR + sh-TESC-1 or sh-ALDH1A1-1 as detected by flow cytometry. In panel **M**–**Q**, *p < 0.05 vs. oe-lncRNA ROR + sh-NC. Cell experiments were repeated three times independently.

The co-expression analysis website MEM predicted the top 500 genes co-expressed with the TESC (Fig. 3A). Differential analysis on the thyroid cancer-related datasets GSE3467, GSE33630, and GSE97001 retrieved from the GEO database revealed 239, 1204, and 1485 differentially expressed genes in thyroid cancer samples relative to normal samples, respectively. Following Venn diagram analysis of the predicted genes, 10 genes were found at the intersection (Fig. 3B and Table S4). These 10 genes were used to construct protein–protein interaction (PPI) network using GeneMANIA (Fig. 3C) to obtain the core degree of these genes, leading to the identification of ALDH1A1 (a top 3 hit) (Table S4). A recent study has reported that TESC promotes the expression of ALDH1A1 in lung cancer [14] and high expression of ALDH1A1 is associated with poor survival of PTC patients [15]. Therefore, we chose ALDH1A1 as the target gene for the follow-up research.

Immunohistochemistry results suggested that relative to adjacent tissues, ALDH1A1 expression in PTC tissues was markedly elevated (Fig. 3D). Additionally, RT-qPCR data displayed that TESC expression was markedly enhanced following oe-TESC treatment, while treatment of sh-TESC-1 or sh-TESC-2 resulted in the opposite outcome in TPC-1 and BCPAP cells (Fig. 3E and Fig. S3A). In addition, the results of RT-qPCR and western blot analysis showed that oe-TESC treatment markedly increased ALDH1A1 expression, while treatment of sh-TESC-1 or sh-TESC-2 contributed to a notable decline in ALDH1A1 expression in TPC-1 and BCPAP cells (Fig. 3F, G and Fig. S3B, C).

Subsequently, ALDH1A1 was overexpressed or silenced in TPC-1 and BCPAP cells, and ALDH1A1 expression in those cells was evaluated using RT-qPCR. As expected in Fig. 3H and Fig. S3D, ALDH1A1 expression displayed a notable increase in the presence of overexpression of ALDH1A1, which was opposite upon treatment of sh-ALDH1A1-1 or sh-ALDH1A1-2. Combined treatment of oe-TESC and sh-ALDH1A1-1 reversed the trend caused by treatment of oe-TESC alone. Subsequently, the effects of ALDH1A1 on the proliferation of PTC cells were assessed by MTT and colony formation assay (Fig. 3I, J and Fig. S3E, F), migration and invasion by Transwell assay (Fig. 3K and Fig. S3G), and apoptosis by flow cytometry (Fig. 3L and Fig. S3H). The results suggested that cell viability, size and number of colonies, migration, and invasion were increased while apoptosis was reduced by lentivirus-mediated overexpression of ALDH1A1 or TESC. However, the opposite results were triggered by sh-ALDH1A1-1 or sh-ALDH1A1-2. The promoting effects of oe-TESC on cell viability, size and number of colonies, migration and invasion as well as inhibitory effects on cell apoptosis were abolished in cells transduced with oe-TESC + sh-ALDH1A1-1. These results demonstrated that TESC promoted PTC cell proliferation, migration, and invasion while inhibiting cell apoptosis by upregulating ALDH1A1.

To verify whether lncRNA ROR could mediate the biological behaviors of PTC cells by regulating the expression of TESC and ALDH1A1, we also overexpressed lncRNA ROR and silenced TESC or ALDH1A1 in TPC-1 and BCPAP cells at the same time. As depicted in Fig. 3M and Fig. S3I, compared with treatment with oe-lncRNA ROR + sh-negative control (NC), treatment of oe-lncRNA ROR + sh-TESC-1 or oe-lncRNA ROR + sh-ALDH1A1-1 resulted in a marked decline in the ALDH1A1 mRNA expression. MTT assay, colony formation assay (Fig. 3N, O and Fig. S3J, K), and Transwell assay (Fig. 3P and Fig. S3L) showed that relative to overexpression of lncRNA ROR alone, both oe-lncRNA ROR and sh-TESC-1 or both oe-lncRNA ROR and sh-ALDH1A1-1, led to significantly reduced cell viability, migration and invasion as well as decreased size and number of colonies. According to flow cytometric analysis, cell apoptosis was promoted in response to transduction with oe-lncRNA ROR + sh-TESC-1 or oe-lncRNA ROR + sh-ALDH1A1-1 (Fig. 3Q and Fig. S3M). Cumulatively, lncRNA ROR activated ALDH1A1/TESC, thereby stimulating PTC cell proliferation, migration, and invasion while inhibiting cell apoptosis.

### ALDH1A1 promoted PTC cell proliferation, migration, and invasion while inhibiting cell apoptosis by increasing TUBB3 expression

**Fig. 4:**
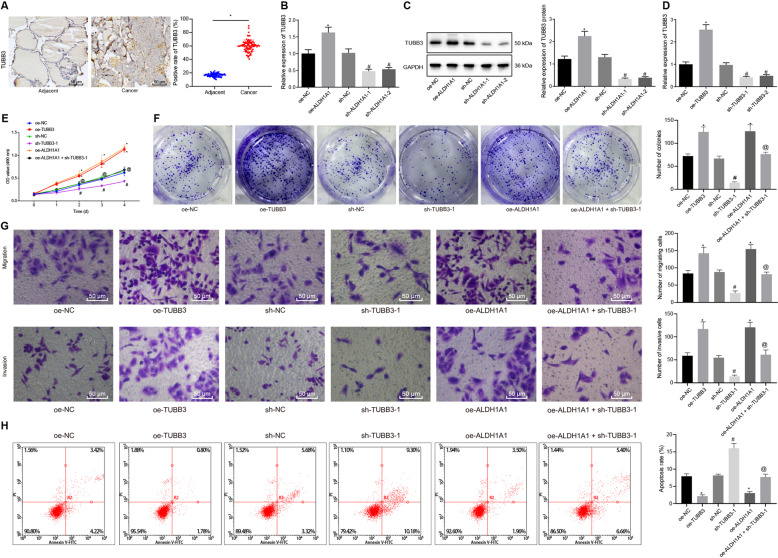
ALDH1A1 activates TUBB3 to facilitate TPC-1 cell proliferation, migration, and invasion while inhibiting cell apoptosis. **A** TUBB3 expression in PTC tissue and adjacent tissue samples of patients with PTC as detected by immunohistochemistry (×400, *n* = 85). *p < 0.05 vs. adjacent tissues. **B** TUBB3 expression after alteration of ALDH1A1 in TPC-1 cells as determined by RT-qPCR, normalized to GAPDH. **C** TUBB3 protein expression after alteration of ALDH1A1 in TPC-1 cells as determined by western blot analysis, normalized to GAPDH. **D** TUBB3 expression after alteration of TUBB3 in TPC-1 cells as determined by RT-qPCR. **E** The effects of TUBB3 on TPC-1 cell viability, as detected by MTT. **F** The effects of TUBB3 on TPC-1 cell colony formation, as detected by colony formation assay. **G** TPC-1 cell migration and invasion after ALDH1A1 overexpression and TUBB3 silencing, as detected by Transwell assay (×200). **H** TPC-1 cell apoptosis after ALDH1A1 overexpression and TUBB3 silencing, as detected by flow cytometry. In panel **B**–**H**, *p < 0.05 vs. oe-NC, ^#^*p* < 0.05 *vs.* sh-NC, ^@^*p* < 0.05 vs. oe-ALDH1A1. Cell ex*p*eriments were repeated three times independently.

It was reported that ALDH1A1 could upregulate TUBB3 to promote bladder cancer progression [16]. Therefore, we speculated that ALDH1A1 might increase TUBB3 expression to regulate PTC progression. To verify this speculation, we firstly assessed TUBB3 expression in PTC tissue and adjacent tissue samples of patients with PTC by immunohistochemistry (Fig. 4A). Compared to adjacent tissues, PTC tissues displayed notably increased expression of TUBB3. Following gain- and loss-of-function of ALDH1A1 in TPC-1 and BCPAP cells, TUBB3 expression was measured using both RT-qPCR and western blot analysis (Fig. 4B, C and Fig. S4A, B). From the results obtained, TUBB3 expression was significantly increased by overexpression of ALDH1A1, but decreased in the presence of sh-ALDH1A1-1 or sh-ALDH1A1-2.

Next, TUBB3 was altered in TPC-1 and BCPAP cells, and RT-qPCR was applied to determine TUBB3 expression in those cells (Fig. 4D and Fig. S4C). As expected, overexpression of TUBB3 resulted in notably increased TUBB3 expression, while silencing of TUBB3 caused the opposite results. Then, PTC cell proliferation was detected using MTT and colony formation assay (Fig. 4E, F and Fig. S4D, E), cell migration and invasion using Transwell assay (Fig. 4G and Fig. S4F), and apoptosis using flow cytometry (Fig. 4H and Fig. S4G). As displayed, cell viability, size and number of colonies, migration, and invasion increased obviously upon oe-TUBB3 treatment, which was diminished after treatment with sh-TUBB3-1. Furthermore, cell viability, size and number of colonies, migration, and invasion displayed a notable elevation in response to lentivirus-mediated overexpression of ALDH1A1, which was abrogated by further silencing of TUBB3. The resulting cell apoptosis changes were found to be opposite. Taken together, ALDH1A1 induced PTC cell malignant phenotypes via upregulating TUBB3.

### LncRNA ROR promoted PTC development and progression through regulation of the TESC/ALDH1A1/TUBB3/PTEN axis

**Fig. 5:**
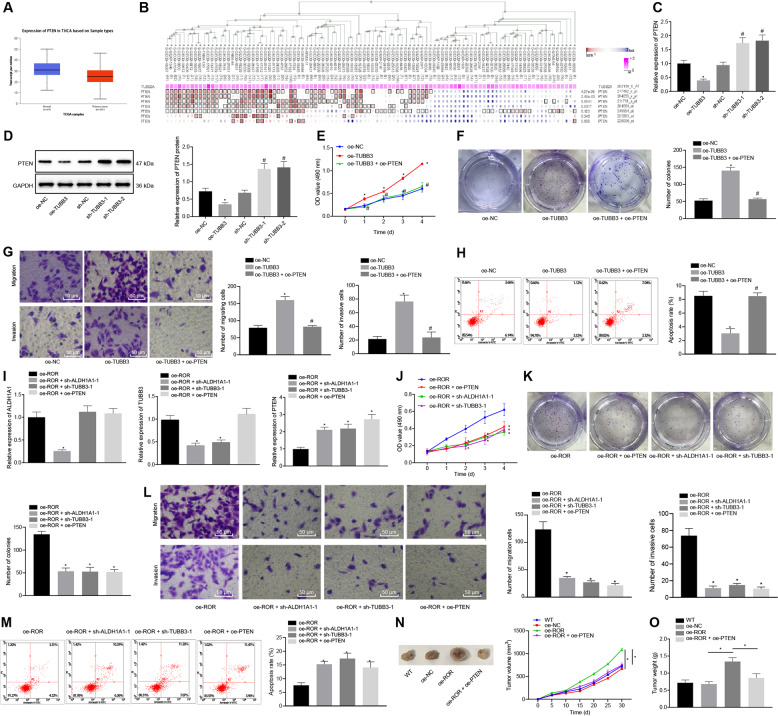
LncRNA ROR promotes the development and progression of PTC through the TESC/ALDH1A1/TUBB3/PTEN axis. **A** A box graph of PTEN expression in the thyroid cancer samples from the TCGA database analyzed using UALCAN. The blue box on the left represents the expression of normal samples, and the red box on the right represents the expression of tumor samples. **B** The relationship between TUBB3 (TUBB2A) expression and PTEN expression through MEM analysis. **C** PTEN expression after TUBB3 alteration in TPC-1 cells as determined by RT-qPCR, normalized to GAPDH. *p < 0.05 vs. oe-NC. ^#^*p* < 0.05 vs. sh-NC. **D** PTEN protein expression after TUBB3 alteration in TPC-1 cells as determined by western blot analysis, normalized to GAPDH. *p < 0.05 vs. oe-NC. ^#^*p* < 0.05 vs. sh-NC. **E** The effects of TUBB3 on TPC-1 cell viability by regulating PTEN, as detected by MTT. **F** The effects of TUBB3 on TPC-1 cell colony formation by regulating PTEN, as detected by colony formation assay. **G** The effects of TUBB3 on TPC-1 cell migration and invasion by regulating PTEN, as detected by Transwell assay (×200). **H** The effects of TUBB3 on TPC-1 cell apoptosis by regulating PTEN, as detected by flow cytometry. In panel **E**–**H**, *p < 0.05 vs. oe-NC. ^#^*p* < 0.05 vs. oe-TUBB3. **I** The expression of ALDH1A1, TUBB3, and PTEN after treatment with oe-lncRNA ROR + sh-ALDH1A1-1, oe-lncRNA ROR + sh-TUBB3-1, or oe-lncRNA ROR + oe-PTEN in TPC-1 cells as determined by RT-qPCR, normalized to GAPDH. **J** The effects of lncRNA ROR on TPC-1 cell viability rate by regulating ALDH1A1, TUBB3, and PTEN, as detected by MTT. **K** The effects of lncRNA ROR on TPC-1 cell colony formation by regulating ALDH1A1, TUBB3, and PTEN, as detected by colony formation assay. **L** The effects of lncRNA ROR on TPC-1 cell migration and invasion by regulating ALDH1A1, TUBB3, and PTEN, as detected by Transwell assay (×200). **M** The effects of lncRNA ROR on TPC-1 cell apoptosis by regulating ALDH1A1, TUBB3 and PTEN, as detected by flow cytometry. In panel **I**–**M**, *p < 0.05 vs. oe-lncRNA ROR. **N** Tumor volume growth curve in mice injected with TPC-1 cells upon lncRNA ROR overexpression or combined with PTEN overexpression (*n* = 12). **O** Tumor weight of mice injected with TPC-1 cells upon lncRNA ROR overexpression or combined with PTEN overexpression (*n* = 12). *p < 0.05 vs. oe-NC or oe-lncRNA ROR. Cell experiments were repeated three times independently.

Based on the analysis from UALCAN, PTEN was found to be poorly expressed in thyroid cancer samples (Fig. 5A). In addition, the co-expression analysis by MEM also showed that TUBB3 (TUBB2A) and PTEN were negatively correlated (Fig. 5B). Western blot analysis revealed that TESC, ALDH1A1, and TUBB3 were highly expressed while PTEN was lowly expressed in TPC-1 and BCPAP cells (Fig. S5A). Following lentivirus-mediated overexpression or silencing of TUBB3 in TPC-1 and BCPAP cells, PTEN expression was determined by RT-qPCR and western blot analysis (Fig. 5C, D and Fig. S5B, C). The results revealed that PTEN expression was significantly declined by lentivirus-mediated overexpression of TUBB3, but enhanced by silencing TUBB3.

MTT and colony formation assays were applied to detect the effects of TUBB3 on PTC cell proliferation by regulating PTEN (Fig. 5E, F and Fig. S5D, E), followed by assessment of cell migration and invasion using Transwell assay (Fig. 5G and Fig. S5F) and cell apoptosis using flow cytometry (Fig. 5H and Fig. S5G). Elevation of cell viability, migration, invasion, and number and size of colonies were observed while cell apoptosis was suppressed in response to TUBB3 overexpression, which was neutralized by simultaneous overexpression of TUBB3 and PTEN. The above results indicated that TUBB3 could promote the proliferation, migration, and invasion of PTC cells and inhibit their apoptosis by inhibiting the expression of PTEN.

To verify whether lncRNA ROR affected PTC cell biological processes through TESC/ALDH1A1/TUBB3/PTEN axis, we overexpressed lncRNA ROR in TPC-1 and BCPAP cells and simultaneously silencing ALDH1A1 or TUBB3 or overexpressing PTEN. ALDH1A1, TUBB3, and PTEN expression was evaluated by RT-qPCR (Fig. 5I and Fig. S5H). Compared with cells transduced with oe-lncRNA ROR, oe-lncRNA ROR + sh-ALDH1A1-1 in cells contributed to significant decreases in terms of the expression of ALDH1A1 and TUBB3 as well as an increase in the expression of PTEN. However, transduction with oe-lncRNA ROR + sh-TUBB3-1 led to no significant changes in the expression of ALDH1A1 or TUBB3 but a marked increase in that of PTEN. Moreover, simultaneous overexpression of lncRNA ROR and PTEN resulted in no significant changes in the expression of ALDH1A1 or TUBB3, but a significant increase in the expression of PTEN. The effects of lncRNA ROR on PTC cell proliferation, migration, invasion, and apoptosis by regulating PTEN were explored using MTT and colony formation assay (Fig. 5J, K and Fig. S5I, J), Transwell assay (Fig. 5L, Fig. S5K), and flow cytometry (Fig. 5M, Fig. S5L). Relative oe-lncRNA ROR treatment, cell viability, migration and invasion as well as size and number of colonies was reduced while cell apoptosis was enhanced by transduction with oe-lncRNA ROR + sh-ALDH1A1-1, oe-lncRNA ROR + sh-TUBB3-1, or oe-lncRNA ROR + oe-PTEN.

In an attempt to characterize the effect of lncRNA ROR and its downstream pathway TESC/ALDH1A1/TUBB3/PTEN axis on PTC in vivo, transduced TPC-1 and BCPAP cells were subcutaneously inoculated into BALB/c nude mice to construct a PTC tumor-bearing model. Tumor growth of mice was observed every 5 days (Fig. 5N and Fig. S5M). After 30 days, the mice were euthanized and the tumor weight of mice was weighed (Fig. 5O and Fig. S5N, O). The results demonstrated that the tumor weight was increased significantly in response to overexpression of lncRNA ROR, which was reversed by further overexpression of PTEN. Taken together, lncRNA ROR accelerated PTC development and progression through regulation of the TESC/ALDH1A1/TUBB3/PTEN axis.

## Discussion

PTC is the most common endocrine malignancy with an increasing incidence yearly [21]. The regulatory roles of several lncRNAs on thyroid cancer have been documented [22]. In the present study, the roles of lncRNA ROR in PTC were investigated. Consequently, our study revealed that lncRNA ROR promotes the cell malignant behaviors and tumor growth of PTC, which is achieved in part by the TESC/ALDH1A1/TUBB3/PTEN axis.

First, we found that lncRNA ROR was highly expressed in PTC tissues and cells. Overexpression of lncRNA ROR significantly increased cell viability, migration, invasion, and colony forming-ability in PTC cells but reduced cell apoptosis. LncRNA ROR has been highlighted as a crucial regulator in multiple cancer types [23] such as hepatocellular carcinoma [24]. In addition, lncRNA ROR was found to be highly expressed in the nuclei of PTC cells while siRNA-mediated silencing of lncRNA ROR resulted in suppressed cell proliferation and invasion [10]. In consistency with our results, lncRNA ROR has been reported to promote viability and proliferation while suppressing apoptosis of esophageal squamous cell carcinoma cells [25]. Likewise, the oncogenic role of lncRNA ROR has been validated in breast cancer by enhancing proliferation and invasion potential of malignant cells in association with dismal oncologic outcomes [26]. Silencing of lncRNA ROR could reverse the promotive effect on liver cancer HepG2 cell proliferation by HepG2 cells-derived exosomes [27].

Another important finding was that lncRNA ROR inhibited the recruitment of G9a to the TESC promoter, thereby upregulating TESC expression. G9a is a lysine methyltransferase that di-methylates H3K9me2. H3K9me2 controls gene silencing via the recruitment of H3K9me2-binding proteins [28]. H3K9 methylation by the methyltransferase G9a results in transcriptional repression of target genes [29]. In the present study, inhibiting G9a recruitment on the promoter of TESC and H3K9me methylation therefore, in turn, caused an increase in the TESC expression. Moreover, lncRNA ROR was previously found to activate the TESC promoter via the suppression of G9a methyltransferase as well as the promotion of histone H3K9 methylation release in tumors, and downregulation of ROR could result in silence of TESC expression [11]. Moreover, our study also observed upregulation of TESC in PTC tissues, and that TESC overexpression promoted PTC cell proliferation, migration, and invasion. TESC was found to be expressed at a high level in pediatric post-chernobyl PTC tumors [13]. In line with the results obtained in our study, knockdown of TESC led to decreased cell migration as well as invasion of colorectal cancer cells [30]. Additionally, silencing of TESC was also reported to suppress cell proliferation and inhibit tumor growth in a xenograft model of colorectal cancer [31]. Largely in agreement with our finding, downregulated TESC induced by specific shRNA has been demonstrated to exert inhibitory effects on renal cell carcinoma cell viability, migration, and invasion [32]. Taken together, we showed that lncRNA ROR promotes the progression of PTC by upregulating TESC expression.

Moreover, we found that the promoting role of TESC in the development of PTC was achieved through upregulation of ALDH1A1 expression and the latter TUBB3. A recent study has shown that STAT3 activation induced by TESC can upregulate the expression of ALDH1 in non-small cell lung cancer [14], suggesting that TESC can also potentially induce the upregulation of ALDH1. Parallel to our finding, ALDH1A1 displayed notably higher expression in PTC tissues than in normal tissues and had a positive correlation with the tumor stage and size [33]. Similarly, ALDH1A1 knockdown resulted in suppressed proliferative and migratory potential of lung cancer cells [34]. Moreover, ALDH1A1 contributed to CCR2-mediated breast cancer cell growth and invasion [35]. Notably, TUBB3 has been documented to be a downstream target of ALDH1A1 and can be positively regulated by ALDH1A1 in bladder cancer [16]. Previously published data have confirmed that treatment with follicle-stimulating hormone can upregulate the expression of TESC and TUBB3 in the hypogonadal mouse simultaneously [36], which indicates the possible positive correlation between TESC and TUBB3. Therefore, disruption of the TESC-mediated ALDH1A1/TUBB3 upregulation can serve as a therapeutic target to prevent PTC.

Furthermore, our results uncovered that ALDH1A1 increased TUBB3 expression to downregulate PTEN, which then promoted PTC cell proliferation, migration, and invasion but suppressed cell apoptosis. In concert with our findings, a previous study found that the mRNA levels of TUBB3 had correlation with lymph nodes status, tumor stages, and molecular markers of breast cancer [37]. Downregulation of TUBB3 was closely correlated with inhibited breast cancer cell proliferation and promoted cell apoptosis [38]. In addition, lack of PTEN led to enhanced MAPK signaling in aggressive thyroid cancer [39]. Moreover, downregulation of TUBB3 contributed to increased PTEN expression, which was beneficial for re-sensitizing docetaxel-resistant prostate cancer cell lines to cabazitaxel [40]. Downregulating TUBB3 by AC10364 suppressed cell viability and proliferation in hepatocellular carcinoma [41].

**Fig. 6:**
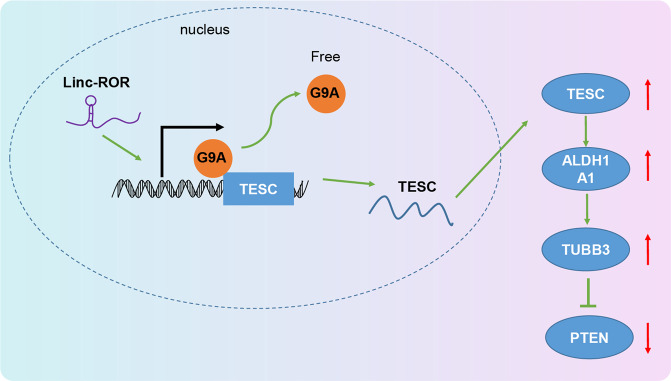
The mechanism of lncRNA ROR/TESC/ALDH1A1/TUBB3/PTEN in the development of PTC. LncRNA ROR activates the TESC/ALDH1A1/TUBB3 axis and consequently leads to downregulation of PTEN, thereby promoting the development of PTC.

To sum up, this present study provided evidence that lncRNA ROR is able to mediate the TESC/ALDH1A1/TUBB3/PTEN axis, thereby promoting the development of PTC (Fig. 6). This finding is very likely to provide a new understanding of the molecular mechanism behind the progression of PTC. In spite of that, a more in-depth investigation is still required to verify its applicable value in clinical practice.

## Supplementary information

supplemental materials

## Data Availability

The datasets generated and/or analyzed during the current study are available in the manuscript and Supplementary Materials.
