# Supplementary material for: Long non-coding ROR promotes the progression of papillary thyroid carcinoma through regulation of the TESC/ALDH1A1/TUBB3/PTEN axis
Source: Cell Death Dis. 2022 Feb 16;13(2):157. doi: 10.1038/s41419-021-04210-9 (PMC8850450; doi:10.1038/s41419-021-04210-9)
Supplement: Supplementary file 1 — supplemental materials [file 41419_2021_4210_MOESM1_ESM.docx]

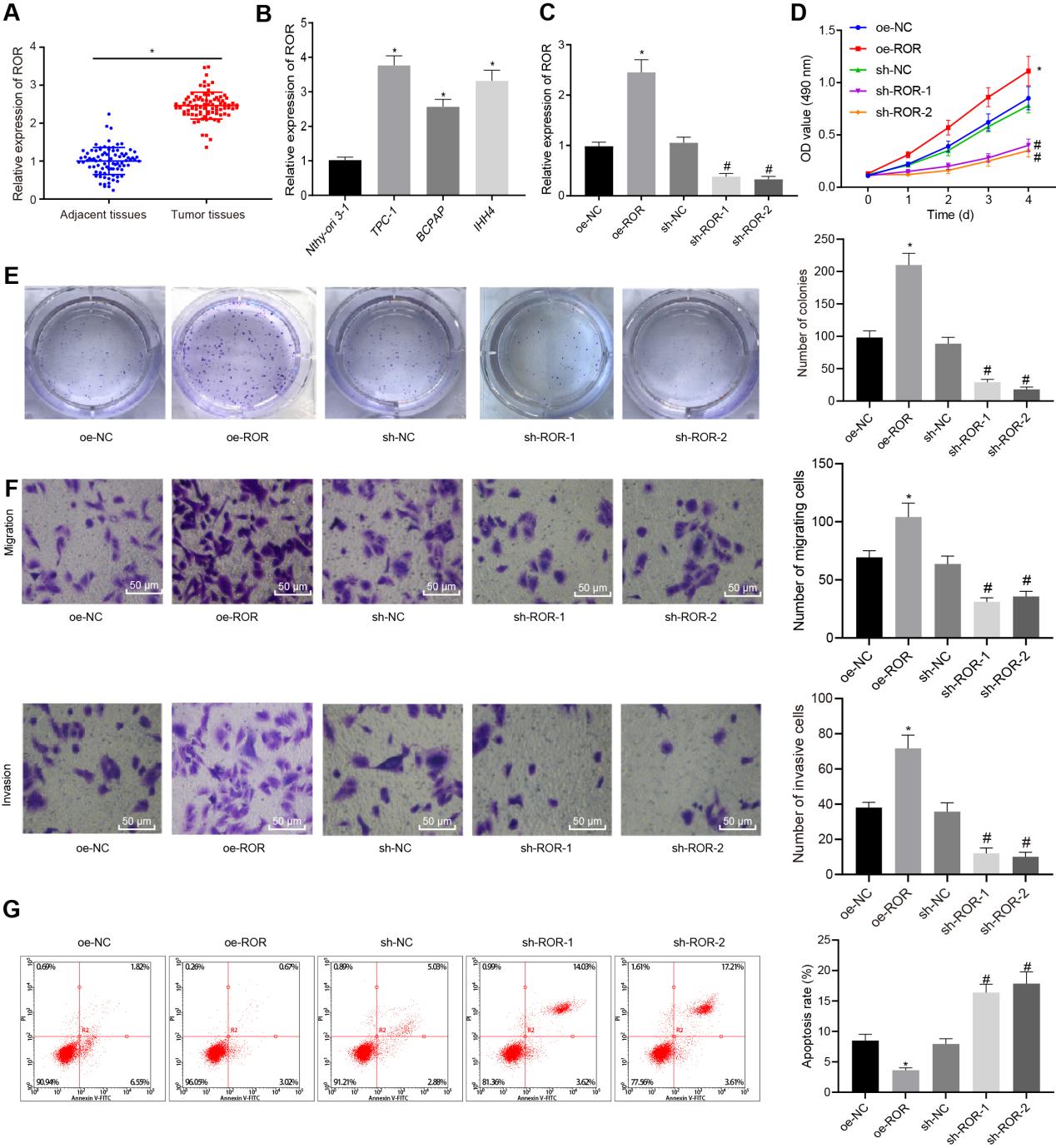


**Figure S1** LncRNA ROR promotes features associated to malignancy in BCPAP cells. A, LncRNA ROR overexpression and silencing efficiency in BCPAP cells verified by RT-qPCR. B, BCPAP cell viability after alteration of lncRNA ROR as detected by MTT. C, Size and number of colonies in BCPAP cells after alteration of lncRNA ROR, as detected by colony formation assay. D, BCPAP cell migration and invasion after alteration of lncRNA ROR as detected by Transwell assay (× 200). E, BCPAP cell apoptosis after alteration of lncRNA ROR as detected by flow cytometry. * *p* < 0.05 *vs*. oe-NC, # *p* < 0.05 *vs*. sh-NC. Cell experiments were repeated three times independently.


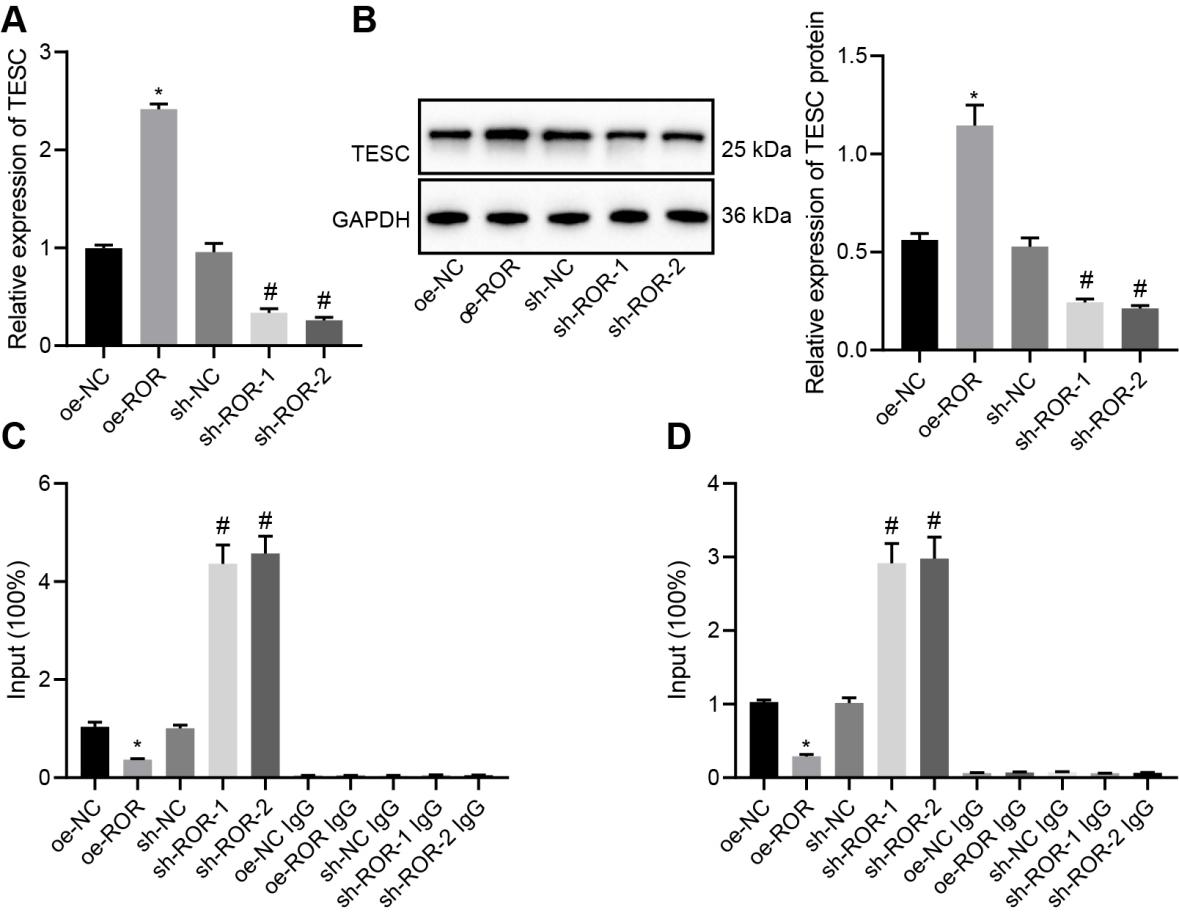


**Figure S2** LncRNA ROR increases TESC expression by repressing the recruitment of G9a on the TESC promoter and H3K9me methylation in BCPAP cells. A, TESC expression after alteration of lncRNA ROR in BCPAP cells as determined by RT-qPCR, normalized to GAPDH. B, TESC protein expression after alteration of lncRNA ROR in BCPAP cells as determined by Western blot analysis, normalized to GAPDH. C, G9a recruitment on the promoter of TESC after alteration of lncRNA ROR in BCPAP cells, as measured by ChIP-PCR. D, H3K9me methylation of the TESC promoter after alteration of lncRNA ROR in BCPAP cells, as detected by ChIP-PCR. * *p* < 0.05 *vs*. oe-NC, # *p* < 0.05 *vs*. sh-NC. The cell experiment was repeated three times independently.


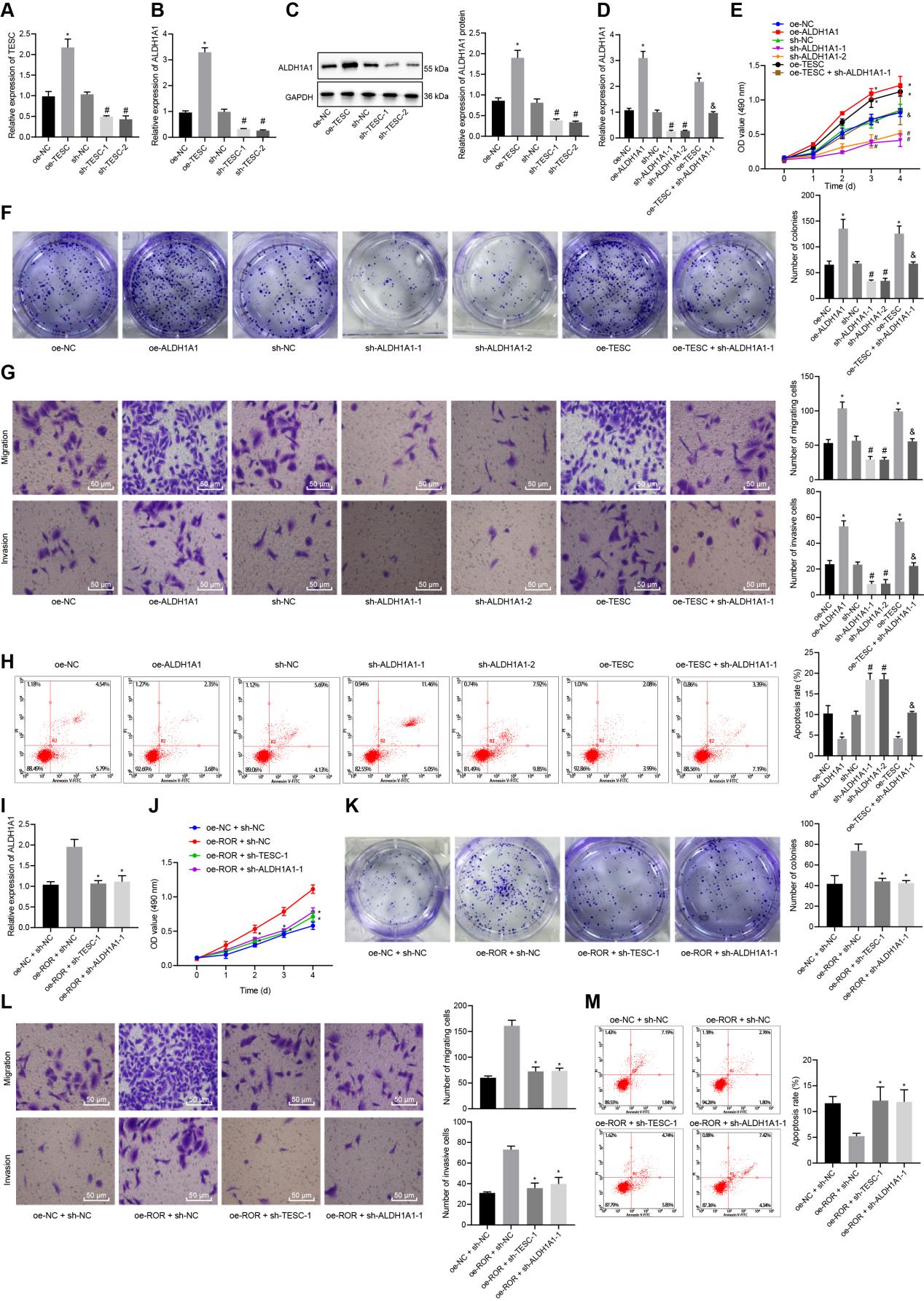


**Figure S3** LncRNA ROR upregulates ALDH1A1/TESC to promote BCPAP cell proliferation, migration and invasion while inhibiting cell apoptosis. A, TESC expression after alteration of TESC in BCPAP cells as determined by RT-qPCR, normalized to GAPDH. * *p* < 0.05 *vs*. oe-NC. # *p* < 0.05 *vs*. sh-NC. B, ALDH1A expression after alteration of TESC in BCPAP cells as determined by RT-qPCR, normalized to GAPDH. * *p* < 0.05 *vs*. oe-NC. # *p* < 0.05 *vs*. sh-NC. C, ALDH1A protein expression after alteration of TESC in BCPAP cells as determined by Western blot analysis, normalized to GAPDH. * *p* < 0.05 *vs*. oe-NC. # *p* < 0.05 *vs*. sh-NC. D, ALDH1A1 expression after ALDH1A1 alteration and TESC overexpression in BCPAP cells as determined by RT-qPCR. E, BCPAP cell viability after ALDH1A1 alteration and TESC overexpression, as detected by MTT. F, Colony formation of BCPAP cells after ALDH1A1 alteration and TESC overexpression, as detected by colony formation assay. G, BCPAP cell migration and invasion after ALDH1A1 alteration and TESC overexpression as detected by Transwell assay (× 200). H, BCPAP cell apoptosis after ALDH1A1 alteration and TESC overexpression as detected by flow cytometry. In panel D-H, * *p* < 0.05 *vs*. oe-NC, # *p* < 0.05 *vs*. sh-NC, & *p* < 0.05 *vs*. oe-TESC. I, The expression of ALDH1A1 in BCPAP cells transduced with oe-lncRNA ROR + sh-TESC-1 or sh-ALDH1A1-1, as determined by RT-qPCR, normalized to GAPDH. J, BCPAP cell viability after transduction with oe-lncRNA ROR and sh-TESC-1 or sh-ALDH1A1-1, as detected by MTT. K, Colony formation of BCPAP cells after transduction with oe-lncRNA ROR + sh-TESC-1 or sh-ALDH1A1-1, as detected by colony formation assay. L, BCPAP cell migration and invasion after transduction with oe-lncRNA ROR + sh-TESC-1 or sh-ALDH1A1-1 as detected by Transwell assay (× 200). M, BCPAP cell apoptosis after transduction with oe-lncRNA ROR + sh-TESC-1 or sh-ALDH1A1-1 as detected by flow cytometry. In panel I-M, * *p* < 0.05 *vs*. oe-lncRNA ROR + sh-NC. The experiment was repeated three times independently.

**
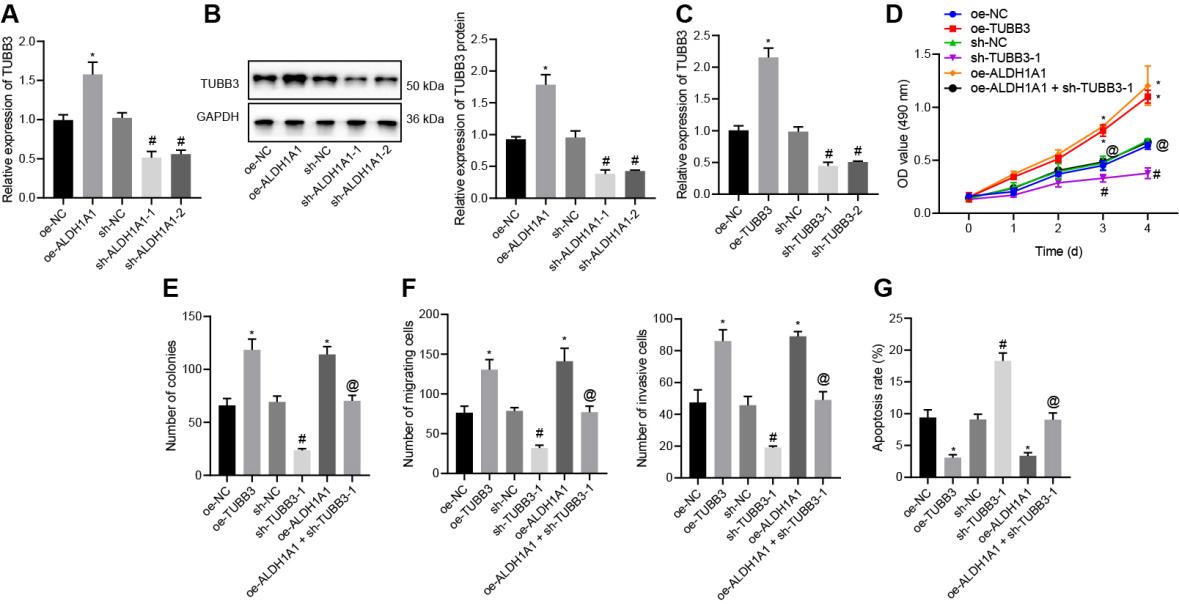
**

**Figure S4** ALDH1A1 activates TUBB3 to facilitate BCPAP cell proliferation, migration and invasion while inhibiting cell apoptosis. A, TUBB3 expression after alteration of ALDH1A1 in BCPAP cells as determined by RT-qPCR, normalized to GAPDH. B, TUBB3 protein expression after alteration of ALDH1A1 in BCPAP cells as determined by Western blot analysis, normalized to GAPDH. C, TUBB3 expression after alteration of TUBB3 in BCPAP cells as determined by RT-qPCR. D, The effects of TUBB3 on BCPAP cell viability, as detected by MTT. E, The effects of TUBB3 on BCPAP cell colony formation, as detected by colony formation assay. F, BCPAP cell migration and invasion after ALDH1A1 overexpression and TUBB3 silencing, as detected by Transwell assay. G, BCPAP cell apoptosis after ALDH1A1 overexpression and TUBB3 silencing, as detected by flow cytometry. * *p* < 0.05 *vs*. oe-NC, # *p* < 0.05 *vs*. sh-NC, @ *p* < 0.05 *vs*. oe-ALDH1A1. Cell experiments were repeated three times independently.

**
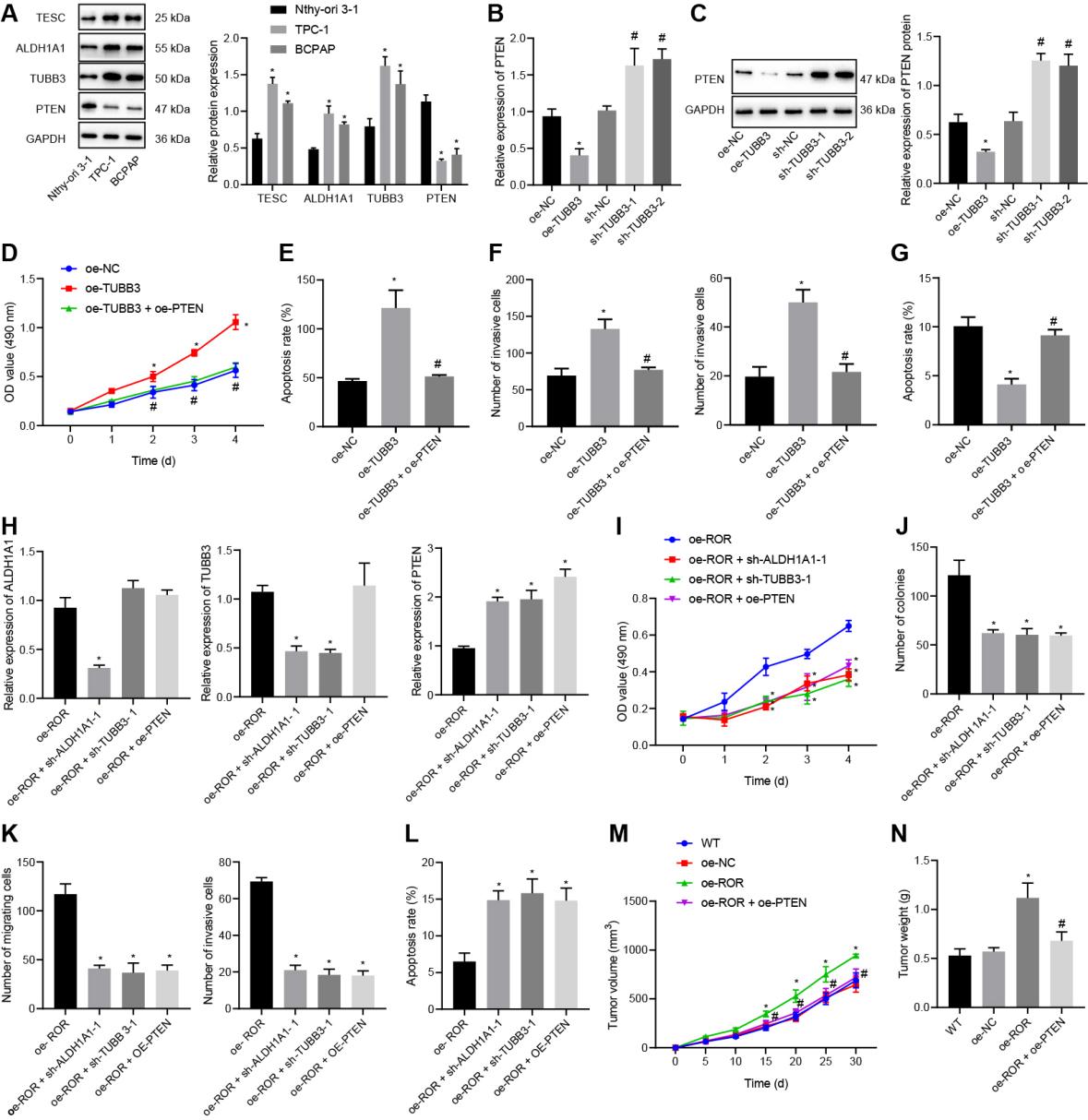
**

**Figure S5** LncRNA ROR promotes the development and progression of PTC through the TESC/ALDH1A1/TUBB3/PTEN axis. A, Endogenous protein expression of TESC/ALDH1A1/TUBB3/PTEN axis in TPC-1 and BCPAP cells determined by Western blot analysis, normalized to GAPDH. B, PTEN expression after TUBB3 alteration in BCPAP cells as determined by RT-qPCR, normalized to GAPDH. * *p* < 0.05 *vs*. oe-NC. # *p* < 0.05 *vs*. sh-NC. C, PTEN protein expression after TUBB3 alteration in BCPAP cells as determined by Western blot analysis, normalized to GAPDH. * *p* < 0.05 *vs*. oe-NC. # *p* < 0.05 *vs*. sh-NC. D, The effects of TUBB3 on BCPAP cell viability by regulating PTEN, as detected by MTT. E, The effects of TUBB3 on BCPAP cell colony formation by regulating PTEN, as detected by colony formation assay. F, The effects of TUBB3 on BCPAP cell migration and invasion by regulating PTEN, as detected by Transwell assay. G, The effects of TUBB3 on BCPAP cell apoptosis by regulating PTEN, as detected by flow cytometry. In panel D-G, * *p* < 0.05 *vs*. oe-NC. # *p* < 0.05 *vs*. oe-TUBB3. H, The expression of ALDH1A1, TUBB3 and PTEN after treatment with oe-lncRNA ROR + sh-ALDH1A1-1, oe-lncRNA ROR + sh-TUBB3-1 or oe-lncRNA ROR + oe-PTEN in BCPAP cells as determined by RT-qPCR, normalized to GAPDH. I, The effects of lncRNA ROR on BCPAP cell viability rate by regulating ALDH1A1, TUBB3, and PTEN, as detected by MTT. J, The effects of lncRNA ROR on BCPAP cell colony formation by regulating ALDH1A1, TUBB3, and PTEN, as detected by colony formation assay. K, The effects of lncRNA ROR on BCPAP cell migration and invasion by regulating ALDH1A1, TUBB3, and PTEN, as detected by Transwell assay. L, The effects of lncRNA ROR on BCPAP cell apoptosis by regulating ALDH1A1, TUBB3, and PTEN, as detected by flow cytometry. In panel H-L, * *p* < 0.05 *vs*. oe-lncRNA ROR. M, Tumor volume growth curve in mice injected with BCPAP cells upon lncRNA ROR overexpression or combined with PTEN overexpression (n = 12). N, Tumor weight of mice injected with BCPAP cells upon lncRNA ROR overexpression or combined with PTEN overexpression (n = 12). * *p* < 0.05 *vs*. oe-NC. Cell experiments were repeated three times independently.

**Table S1** Correlation between lncRNA ROR expression and clinical characteristics of patients with PTC (n = 85)

| Indexes | n = 85 | High expression group  (n = 42) | Low expression group  (n = 43) | *p* |
| --- | --- | --- | --- | --- |
| Gender |  |  |  | 0.279 |
| Male | 20 | 12 (60.00%) | 8 (40.00%) |  |
| Female | 65 | 30 (46.15%) | 35 (53.85%) |  |
| Age (years) |  |  |  | 0.717 |
| < 55 | 78 | 39 (50.00%) | 39 (50.00%) |  |
| ≥ 55 | 7 | 3 (42.86%) | 4 (57.14%) |  |
| Extraglandular invasion |  |  |  | 0.165 |
| Yes | 15 | 10 (66.67%) | 5 (33.33%) |  |
| No | 70 | 32 (42.86%) | 38 (57.14%) |  |
| Lymph node metastasis |  |  |  | 0.751 |
| Yes | 39 | 20 (51.28%) | 19 (48.72%) |  |
| No | 46 | 22 (47.83%) | 24 (52.17%) |  |
| Multifocal cancer |  |  |  | 0.292 |
| Yes | 22 | 13 (59.09%) | 9 (40.91%) |  |
| No | 63 | 29 (46.03%) | 34 (53.97%) |  |
| Tumor diameter |  |  |  | 0.017 |
| < 1 | 31 | 10 (32.26%) | 21 (67.74%) |  |
| ≥ 1 | 54 | 32 (59.26%) | 22 (40.74%) |  |

Note: Low/high expression was identified by being compared with the median of the sample.

**Table S2 Primer Sequences of RT-qPCR.**

| Gene | Sequences |
| --- | --- |
| LncRNA ROR [human] | F: 5´-CTCAGTGGGGAAGACTCCAG-3´ |
|  | R: 5´-AGGAAGCCTGAGAGTTGGC-3´ |
| TESC [human] | F: 5´-CCTACCATTCGGAACCTGCG-3´ |
|  | R: 5´-AGCTCCTCGACCACATTTCG-3´ |
| ALDH1A1 [human] | F: 5´-CTGTGTTCCAGGAGCCGAAT-3´ |
|  | R: 5´-AGCATCCATAGTACGCCACG-3´ |
| TUBB3 [human] | F: 5´-GCTCAGGGGCCTTTGGACATCTCTT-3´ |
|  | R: 5´-TTTTCACACTCCTTCCGCACCACATC-3´ |
| PTEN [human] | F: 5´-ACCCACCACAGCTAGAACTT-3′ |
|  | R: 5´-GGGAATAGTTACTCCCTTTTTGTC-3′ |
| GAPDH [human] | F: 5´-CACCCACTCCTCCACCTTTG-3′ |
|  | R: 5´-CCACCACCCTGTTGCTGTAG-3′ |

**Table S3 Primer Sequences of ChIP-PCR.**

| Gene | Sequences |
| --- | --- |
| TESC [human] | F: 5´-AGGTAAAGACATCTCAAAAGGCCAATC-3´ |
|  | R: 5´-GGCCTAAATTTCGCTAAGACGC-3´ |
| GAPDH [human] | F: 5´-CACCCACTCCTCCACCTTTG-3′ |
|  | R: 5´-CCACCACCCTGTTGCTGTAG-3′ |

**Table S4 Core degree of each gene in the PPI network.**

| Gene | Degree |
| --- | --- |
| SERPINA1 | 36 |
| HGD | 34 |
| ALDH1A1 | 14 |
| AGR2 | 12 |
| METTL7B | 8 |
| KCNN4 | 6 |
| PDZK1IP1 | 6 |
| CTSH | 4 |
| CSGALNACT1 | 1 |
| TMC6 | 0 |
